# The effects of transitioning from immediate release to extended release cysteamine therapy in Norwegian patients with nephropathic cystinosis: a retrospective study

**DOI:** 10.1007/s00467-023-06005-w

**Published:** 2023-05-23

**Authors:** Anna Bjerre, Sonja Amdal Aase, Maria Radtke, Christian Siva, Helga Gudmundsdottir, Brita Forsberg, Berit Woldseth, Damien Brackman

**Affiliations:** 1 Department for Specialised Paediatrics, Oslo University Hospital, Oslo, Norway; 2 Institute of Clinical Medicine, University of Oslo, Oslo, Norway; 3 Department of Paediatric and Adolescent Medicine, Stavanger University Hospital, Stavanger, Norway; 4 Department of Nephrology, St Olav’s University Hospital, Trondheim, Norway; 5 Department of Clinical and Molecular Medicine, Norwegian University of Science and Technology, Trondheim, Norge; 6 Paediatric Department, Vestfold Hospital, Tønsberg, Norway; 7 Nephrology Department, Ullevål, Oslo University Hospital, Oslo, Norway; 8 Chiesi Global Rare Diseases, Nordics, Stockholm, Sweden; 9 Department of Medical Biochemistry, Oslo University Hospital, Oslo, Norway; 10 Children and Adolescents Clinic, Haukeland University Hospital, Bergen, Norway

**Keywords:** Nephropathic cystinosis, Immediate-release cysteamine, Extended-release cysteamine, Lysosomal storage disease, Growth, Kidney function

## Abstract

**Background:**

Nephropathic cystinosis is a rare lysosomal storage disorder in which accumulation of cystine and formation of crystals particularly impair kidney function and gradually lead to multi-organ dysfunction. Lifelong therapy with the aminothiol cysteamine can delay the development of kidney failure and the need for transplant. The purpose of our long-term study was to explore the effects of transitioning from immediate release (IR) to extended release (ER) formulation in Norwegian patients in routine clinical care.

**Methods:**

We retrospectively analysed data on efficacy and safety in 10 paediatric and adult patients. Data were obtained from up to 6 years before and 6 years after transitioning from IR- to ER-cysteamine.

**Results:**

Mean white blood cell (WBC) cystine levels remained comparable between the different treatment periods (1.19 versus 1.38 nmol hemicystine/mg protein) although most patients under ER-cysteamine underwent dose reductions. For the non-transplanted patients, the mean estimated glomerular filtration rate (eGFR) change/year was more pronounced during ER-treatment (− 3.39 versus − 6.80 ml/min/1.73 m^2^/year) possibly influenced by individual events, such as tubulointerstitial nephritis and colitis. Growth measured by Z-height score tended to develop positively. Four of seven patients reported improvement of halitosis, one reported unchanged and two reported worsened symptoms. Most adverse drug reactions (ADRs) were of mild severity. One patient developed two serious ADRs and switched back to IR-formulation.

**Conclusions:**

The results from this long-term retrospective study indicate that switching from IR- to ER-cysteamine was feasible and well tolerated under routine clinical practice. ER-cysteamine allowed satisfactory disease control over the long period considered.

**Graphical abstract:**

**Figure:**
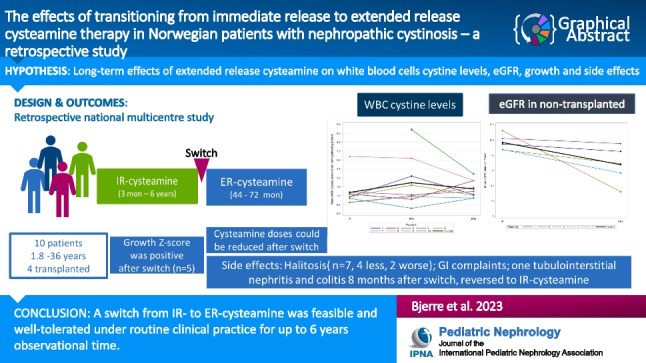
A higher resolution version of the Graphical abstract is available as Supplementary information

**Supplementary information:**

The online version contains supplementary material available at 10.1007/s00467-023-06005-w.

## Introduction

Nephropathic cystinosis is an autosomal recessive lysosomal storage disease in which the cystine transporter cystinosin is impaired due to mutations in the *CTNS* gene. The disease affects one in 100,000 to 200,000 live births and is found in all ethnic groups with a male:female ratio of 1–1.4:1 [1–3]. A lack of function of cystinosin leads to accumulation of free cystine in the lysosomes resulting in an intracellular cystine crystal formation throughout the body leading to organ and tissue damage [1, 2, 4, 5].

About 95% of patients have the infantile nephropathic form of cystinosis [3]. The earliest manifestations of the disease are failure to thrive, rickets and Fanconi syndrome with gradual impairment of kidney function. If treatment is not initiated early enough, kidney failure necessitates dialysis or kidney transplant by the end of the first decade of life. Over time, most organs are affected including the cornea, gastrointestinal tract, muscles and central nervous system [1–3, 5].

Diagnosis of cystinosis is confirmed by measuring elevated white blood cell (WBC) cystine levels and molecular testing of the *CTNS* gene. In healthy individuals, WBC cystine levels can reach 0.2–0.6 nmol hemicystine/mg protein whereas patients generally have values of 3.0–23.0 nmol hemicystine/mg protein [3, 5, 6].

Cysteamine is currently the only cystine depletion therapy approved for nephropathic cystinosis. This aminothiol reacts within lysosomes to convert cystine into cysteine and cysteine-cysteamine-mixed disulphide, both of which can exit the lysosome. Consequently, cysteamine treatment reduces accumulation of cystine and subsequent formation of cystine crystals in the lysosomes [5]. The cysteamine treatment is titrated based on tolerance and the therapeutic goal of maintaining WBC cystine levels below 1 nmol hemicystine/mg of total protein [7–9]. When initiated early in life, it has shown to improve growth, preserve kidney and extra-kidney organ function, and ultimately prolong life expectancy [1, 2, 10]. As the treatment is lifelong, continuous adherence is critical to ensure optimal disease control [12].

An immediate release (IR) formulation of cysteamine is available since 1997 (Cystagon®, Recordati Rare Diseases, Puteaux, France). Although being effective in preserving kidney function, the long-term adherence to the IR-formulation is challenging due to its strict 6-h administration schedule (including one administration during the night) with reported adherence to IR-cysteamine ranging from 23 to 34% [1, 11, 12]. In addition, many patients display very challenging side effects including gastric effects (ulcerogenesis) as well as halitosis and body odour [1, 7, 12–14].

An extended release (ER) formulation of cysteamine for administration every 12 h was approved in 2013 in the USA and in 2014 in Europe (Procysbi®, Horizon Therapeutics, USA, and Chiesi Farmaceutici S.p.A, Parma, Italy). The pivotal study, and its open-label follow-up, demonstrated that the efficacy of ER-cysteamine was non-inferior compared to IR-cysteamine, with fewer long-term adverse events (AEs) and no unexpected adverse drug reactions (ADRs). Moreover, ER-cysteamine was associated with an improved quality of life and a trend towards reduced halitosis caused by cysteamine metabolites [15, 16]. Also, a retrospective real-life single-centre study showed that the switch from IR-cysteamine to ER-cysteamine in twelve paediatric patients was safe and effective over the short-term and indicated less halitosis [6].

Our retrospective, multicentre study assessed the efficacy and safety of oral IR- and ER-cysteamine treatment in a Norwegian patient population. The aim was to evaluate the implementation of the ER-cysteamine therapy in cystinosis patients already treated with IR-cysteamine and to assess the outcomes of this option in routine care.

## Methods

### Study design/patients

**Fig. 1:**
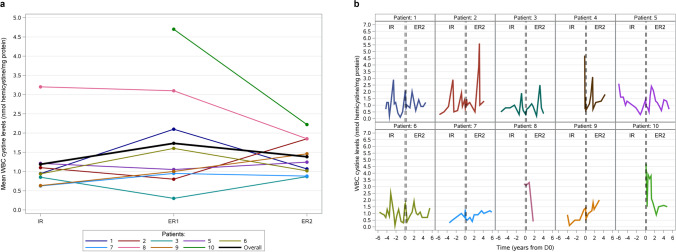
**a** Patient individual means and overall mean of white blood cell (WBC) cystine levels during immediate release (IR) and extended release (ER1 and ER2) treatment periods for patients included in the efficacy analysis. Due to the known non-compliance to the cysteamine dosage, no measurements were performed for patient 10 during IR-treatment. **b** Development of the individual white blood cell (WBC) cystine levels of the 10 Norwegian patients resulting from all available single measurements over time before and after the switch day (D0)

In our non-interventional study, the decision to prescribe IR- or ER-cysteamine therapy was taken prior to, and independently from, the decision to enrol the patients into the study. All patients were under a regular cysteamine therapy. The switch from IR- to ER-treatment was made according to good clinical practice and intentionally for the betterment of the patients. Eligible nephropathic cystinosis patients were identified and screened at six Norwegian study centres. All 10 Norwegian paediatric and adult patients fulfilled the inclusion criteria. Informed consent was obtained from all patients and/or from the patient’s legal representative during the screening period prior to collection of data from the patient medical records. Data were analysed primarily based on IR- and ER-treatment periods before and after the switch. The IR-treatment period was defined as the period from initiation of IR-cysteamine to the switch day (D0), including at least 3 months and a maximum of 6 years. To account for dose adjustments, ER-treatment period 1 (ER1) was defined as the period from the switch day to 3 months after switching minus one day. ER-treatment period 2 (ER2) was defined as the period from 3 months after switching to the time of inclusion in the study or a switch back to IR-cysteamine. ER2 was included in both efficacy and safety evaluations, whilst ER1 was only included in the safety evaluations. For illustrative purposes, ER1 data has been included in Figs. 1a and 4, even though not used in the calculations. Data were collected retrospectively from patient journals and entered into the standardised electronic case report form (eCRF).

### Measurement of WBC cystine levels and eGFR values

Cystine levels in WBCs, measured at all available clinic visits, were obtained from all treatment periods. The number of measurements during each period differed from patient to patient. Individual mean values were thus calculated for each patient for each period. The overall mean WBC cystine values were used to determine the relative and absolute change from the IR- to the ER2-treatment period. Furthermore, the percentages of measured WBC cystine levels ≤ 1 nmol and ≤ 3 nmol of hemicystine per mg protein were compared. For all patients, cystine levels were determined by the cystine binding protein assay until April 2015 and liquid chromatography-tandem mass-spectrometry (LC–MS/MS) from May 2015 by the same laboratory. Before changing the method for analysis, a cross-validation was performed to assure comparability between the assays. Levels of cystine in WBCs were recorded as nmol of hemicystine per mg protein.

Similarly, all available serum creatinine values during the treatment periods were obtained from patient journals and eGFR was thereafter calculated using the CKD-EPI40 equation (Kidney Disease Epidemiology Collaboration equation) with age-adjusted creatinine values for all patients [17]. Difference in mean eGFR change per year between the IR-treatment period and the ER2-treatment period was included as a primary efficacy endpoint to evaluate any significant changes in kidney function after the switch.

### Other measurements

For a comparison of growth between IR- and ER2-treatment periods, the height *Z*-score was calculated using the actual height of the patient and the mean and standard deviation height of a healthy reference population of the same age from the World Health Organization (WHO). Height was recorded up to the age of 19 years.

All available data of total prescribed daily cysteamine doses were collected from all treatment periods. Total prescribed daily cysteamine dose was recorded as mg/day. Based on the individual height and weight of the patients, the doses were normalised to the body surface and expressed as mg/m^2^/day. For patients consenting separately, the dose dispensed at the pharmacy was retrieved from the Norwegian Prescription Database (NorPD). The percentage of the prescribed dose that was actually issued could thus be calculated to estimate adherence. These data were neither entered into the eCRF nor made available to the investigators to secure patient integrity.

Other patient characteristics were extracted from patient records such as the usage of growth hormone and proton pump inhibitors (PPI), the onset of puberty, gastrostomy and time of kidney transplant.

### Safety

The following safety endpoints were collected: halitosis during ER-treatment relative to IR-treatment as judged by the patient and/or caregiver, reported non-serious and serious ADRs, ADRs/SADRs leading to treatment discontinuation and ADRs/SADRs leading to emergency room visits and/or hospitalisation, AEs related to the underlying disease leading to emergency room visits and/or hospitalisations and incidence of IR- and ER-cysteamine treatment discontinuation.

### Analysis

Owing to the small sample size in the study, no formal statistical analyses have been conducted. All data collected were assessed descriptively.

### Consent of local ethic board

The Norwegian Ethics Committees assessed the project to be a quality assurance project with no need for approval. Since the patients and/or caregivers voluntarily signed the informed consent, approval from the Norwegian Directorate of Health was not required either.

## Results

### Baseline characteristics

The patient collective comprised 4 female and 6 male patients, both children and adults. The age at IR- to ER-treatment switch ranged from 1.8 to 36.0 years with a mean age (SD) of 16.09 (± 9.66) years. Two patients had not reached puberty at the time of data collection. Four had undergone kidney transplantation prior to the defined treatment periods and patient 10 received a second transplant during the IR-treatment period. Patient 4 was excluded from the efficacy analysis as his IR-treatment period was shorter than 3 months. Individual patient data are summarised in Table 1.

**Table 1:** Summary of individual patient data

No	Sex	Age at switch (years)	Months on ER-treatment	Puberty onset before/after switch	Age at kidney transplantation***	Use of growth hormone	Use of PPI	Gastrostomy	Halitosis	Reasons for switch
1	M	6.2	61	Prepubertal	No	Yes	No	No	Not reported	Not specified
2	M	19.4	54	Before	7	Yes	No	Yes	Not reported	Non-compliance with IR cysteamine, e.g., difficulties with night-time administration
3	F	19.7	58	Before	7	No	No	No	Not reported	Non-compliance with IR cysteamine, e.g., difficulties with night-time administration
4*	M	1.8	53	Prepubertal	No	No	Yes	Yes	Worsened	Best treatment for patient
5	M	9.7	62	After	No	Yes	No	Yes	Improved	Dosage twice daily
6	F	12.1	66	After	No	Yes	No	Yes	Improved	Non-compliance with IR cysteamine, e.g., difficulties with night-time administration
7	M	16.7	72	Before	No	Yes	Yes	Yes	Unchanged	Dosing twice daily and hoping for fewer side effects, especially nausea and stomach pain
8	F	23.8	64	Before	14	No	Yes	No	Improved	Non-compliance with IR cysteamine, e.g., difficulties with night-time administration
9**	F	15.6	44	Before	No	Yes	No	No	Worsened	IR to ER: Avoid night-time dosing and hope of less halitosis ER to IR: relevant side effects
10	M	36.0	59	Before	9 33	Yes	Yes	No	Improved	Relevant side effects

^*^Not included in efficacy analysis due to short time on IR-treatment before switch

^**^Switched back to IR-treatment in 2019 due to an adverse drug reaction (colitis)

^***^All transplantations took place before the switch

### WBC cystine levels

Individual mean WBC cystine levels during ER2-treatment ranged within the values for IR-treatment in most of the 9 patients included in the efficacy analysis. The overall mean level of WBC cystine remained stable between IR- and ER2-treatment period with 1.19 and 1.38 nmol hemicystine/mg protein, respectively, resulting in an absolute change of 0.09 (± 0.66, median 0.10) (Figs. 1a and b). For patients 8 and 10 with the initially highest numerical individual mean values, WBC cystine level remarkably declined during the ER2-treatment. For patient 10, no WBC cystine values were available during the IR-treatment period. Due to the known non-compliance to the dosage and the consequent conclusion that the WBC cystine levels were too high, no measurements were performed for the patient during this period. When comparing the percentage of measured WBC cystine levels ≤ 1 nmol hemicystine/mg protein, there was a trend towards slightly increased numbers during ER2- compared to IR-treatment (63.22% (± 31.21) versus 47.52% (± 23.71)). However, all 9 patients had at least intermittently WBC cystine levels ≤ 1 nmol during ER2-treatment compared to 8 patients on IR-treatment. The percentage of measurements with WBC levels ≤ 3 nmol hemicystine/mg protein remained almost equal between the periods (87.50% (± 35.36) during IR-treatment versus 88.89% (± 18.33) during ER2-treatment).

### Estimated glomerular filtration rate

**Fig. 2:**
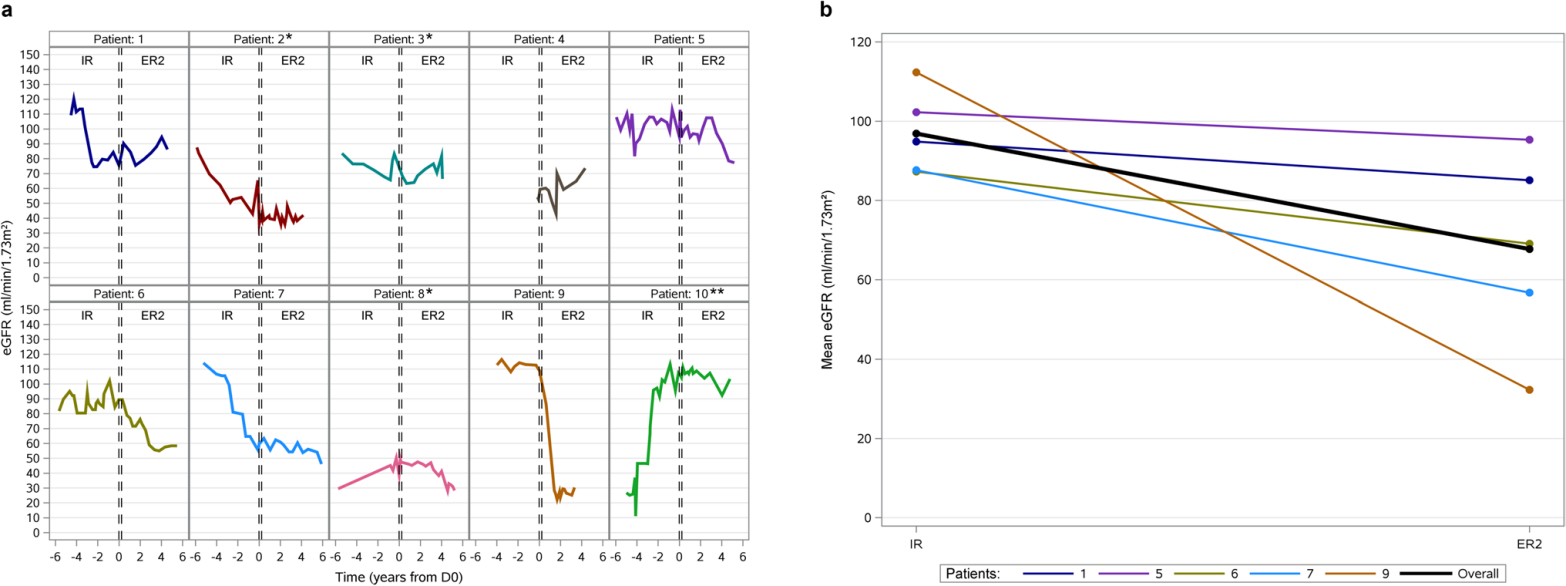
**a** Development of patient individual glomerular filtration rates (eGFR) resulting from all available single measurements for the 10 Norwegian patients over time before and after the switch day (D0). *Patients with one kidney transplantation. **Patient with two kidney transplantations. **b** Patient individual means and overall mean of estimated glomerular filtration rate (eGFR) during immediate release (IR) and extended release (ER2) treatment periods for the non-transplanted patients included in efficacy analysis

In the majority of patients, eGFR gradually decreased over time (Fig. 2a), with individual patients experiencing fluctuations in eGFR caused by distinct individual events. For patient 9, there was a pronounced drop from the IR- to the ER2-treatment period which was associated with the occurrence of colitis and tubulointerstitial nephritis leading to reduced kidney function. On the other hand, for patient 10, the eGFR increased noticeably during the IR-treatment period due to a second kidney transplantation which led to improvement in the filtration rate. For patient 4, who was not included in efficacy analysis due to a short time on IR-treatment before the switch, eGFR increased notably in the ER2-treatment period. This patient started cysteamine treatment early in childhood, at the age of 1.8 years and was quickly switched from IR- to ER-treatment (Fig. 2a).

The individual and overall mean eGFR, as well as the mean eGFR change per year, were determined for the non-transplanted patients. The overall mean eGFR for these 5 patients dropped from 96.86 (± 10.60) ml/min/1.73 m^2^, during the IR-treatment period, to 67.68 (± 24.71) ml/min/1.73 m^2^ during ER2-treatment period (Fig. 2b). The eGFR deteriorated faster during the ER2-treatment period with a mean eGFR change per year of − 3.39 (± 5.31) ml/min/1.73 m^2^/year during IR-treatment and − 6.80 (± 8.25) ml/min/1.73 m^2^/year during ER2-treatment.

### Growth

**Fig. 3:**
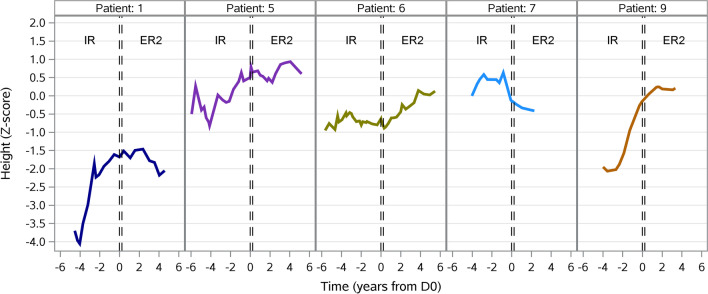
Development of patient individual height *Z*-score resulting from all available single measurements over time before and after the switch day (D0) for the 5 patients still growing during defined treatment periods

The height of the patients was recorded only until the age of 19 years. We determined the development of the height *Z*-score from IR-treatment period to ER2-treatment period for the 5 patients (patients 1, 5, 6, 7, 9) who were still growing during both periods. Changes in individual height *Z*-scores, before and after conversion, are shown in Fig. 3. The overall mean height *Z*-score was − 1.0 (± 1.1) in the IR-treatment period, and − 0.3 (± 0.9) in the ER2-treatment period. Patients 5, 6, 7, 9 used growth hormones in both treatment periods whilst patient 1 started growth hormone treatment during the ER2-treatment period (Table 1).

### Cysteamine doses

**Fig. 4:**
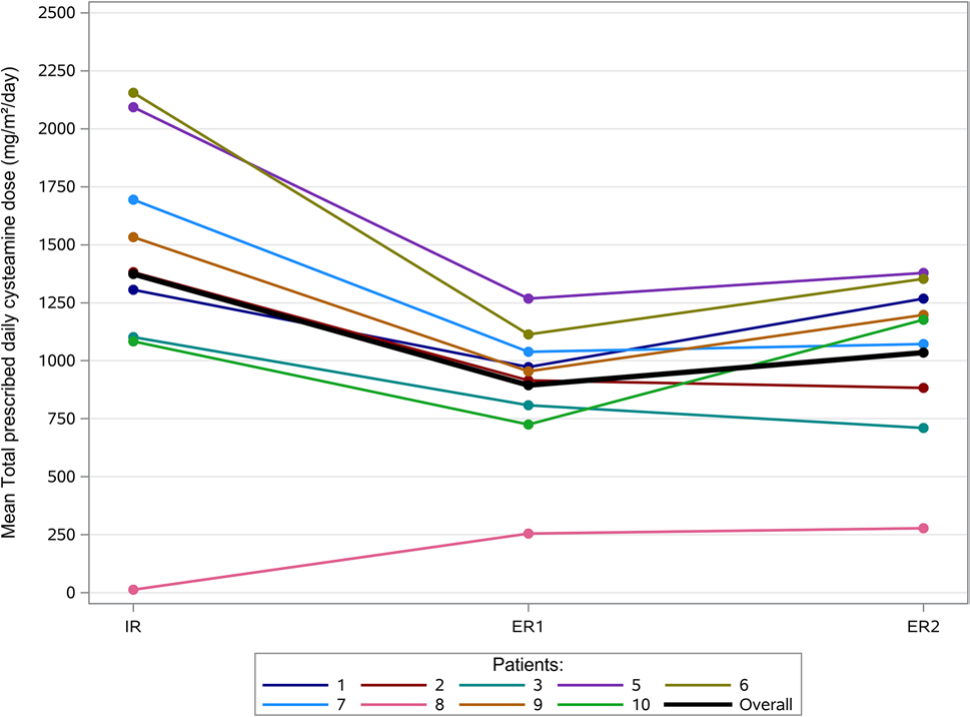
Patient individual means and overall mean of the total prescribed daily cysteamine dose during immediate release (IR) and extended release (ER1 and ER2) treatment periods for patients included in efficacy analysis

For most of the patients the individual mean values of prescribed daily cysteamine doses decreased comparing the IR-treatment period to the ER2-treatment period (Fig. 4). Altogether, the mean total dose could be reduced with the ER-formulation from 1374 (± 640, median 1383) to 1035 (± 358, median 1176) mg/m^2^/day after the switch.

Furthermore, we were able to obtain data on dispensed doses at the pharmacy from the NorPD for 7 patients. The mean percentage of prescribed doses issued from the pharmacy indicate a slightly higher adherence to ER-cysteamine (95.99% versus 99.52%, respectively), which can also be seen from a minimum value of dispensed dose versus prescribed dose of 51% during the ER2-treatment period compared to only 35.1% during the IR-treatment period.

### Other patient characteristics

Data on the usage of growth hormones and PPIs, as well as on the need for gastrostomy are shown in Table 1. For these characteristics, the number of affected patients was too small to allow a reasonable interpretation of the data.

### Safety

For the safety analysis data, all 10 patients were evaluated considering all treatment periods (IR, ER1 and ER2). The total number of ADRs reported was higher with ER-cysteamine (*n* = 12) than with IR-cysteamine (*n* = 4) affecting 6 and 3 patients, respectively. ADRs were mostly of mild to moderate severity (11 and 3 events, respectively). One patient (patient 9) was diagnosed with two SADRs (colitis and tubulointerstitial nephritis) 8 months after the transition. At the time of the SADRs, it was not clear if the colitis could have a relationship with the drug, so the investigator decided to permanently discontinue the ER-cysteamine and switch back to IR-treatment. The most common ADRs were of gastrointestinal origin. Expressed in terms of specific symptoms, halitosis and nausea were experienced each by two patients whereas colitis and constipation were experienced each by one patient only during ER-treatment. Likewise, all other ADRs reported under ER-treatment occurred only once (fatigue, arthralgia, headache, cutis laxa, skin striae). No AEs, related to the underlying disease leading to emergency room visits and/or hospitalisations, occurred during any of the treatment periods.

### Halitosis

A total of 7 patients experienced halitosis some time during the treatment with cysteamine (Table 1). Following the switch to the ER-formulation, 4 patients (57.1%) reported an improvement, one patient (14.3%) reported an unchanged status, and 2 patients (28.6%) reported worsening of symptoms.

### Treatment discontinuation

In this study, we defined cysteamine treatment discontinuation as any occurrence of more than 7 consecutive days without any oral cysteamine administration. Discontinuation was rare during both treatment periods (2 patients in the IR-period versus 3 patients in the ER-period). Only one patient interrupted significantly more often, and longer, for different reasons. For this patient, not only the side effects but also psychological reasons played a role.

## Discussion

In this national retrospective study, all Norwegian patients diagnosed with nephropathic cystinosis switched from the IR- to the ER-formulation during a one-year period as the medication was approved in 2014. For 9 of the 10 patients, treatment with ER-cysteamine is still ongoing and is generally well tolerated. To our knowledge, our study is the longest follow-up after switching from IR- to ER-cysteamine with a median follow-up time of 60 (range 44–72) months.

Ahlenstiel-Grunow et al. published the first retrospective real-life follow-up study analysing the impact of switching on cystinosis patient outcomes. The study, with a median follow-up time of 14 (range 3–18) months, showed that the switch from IR- to ER-cysteamine in twelve paediatric patients was safe and effective over the short-term, providing benefits in terms of frequency of administration and less halitosis [6]. In line with the short-term real-life study, as well as the approval and follow-up trials [6, 15, 16], the ER-formulation in our study was comparable to the IR-formulation in maintaining the WBC cystine levels in patients with cystinosis, with a slight trend towards a lower percent of cystine levels < 1 nmol during the ER-treatment.

Not all patients in our study were well controlled, as defined by WBC cystine levels, before transition to ER-cysteamine, especially those known to be non-adherent with the strict 6-h-based IR-cysteamine dose regimen. However, these patients appeared to benefit from the ER-formulation as their cystine levels dropped to appropriate lower values.

Overall, cystinosis could be adequately controlled in our patients during the long-term treatment with ER-cysteamine. It is worth noting that this was achieved with a lower mean medication requirement consistent with findings reported in previous publications [16, 18]. The authors of these studies considered 60–80% of ER-cysteamine of the total daily dose of IR-cysteamine dosing as being sufficient. In contrast, a recent study analysing cystine levels of patients after a single dose of IR-cysteamine, as well as a single dose of ER-cysteamine, comes to a different conclusion [19]. Based on pharmacokinetic parameters, van Stein et al. recommend starting ER-treatment at a higher dosage than 70% of the previous cysteamine dosage. In addition, they suggest dividing the total dose to three times daily intake, instead of twice daily, to prevent a rapid drop and achieve a steadier decline in cystine levels [19].

As expected, due to the long medium observation period of approximately 9.5 years, and the natural disease progression in the non-transplanted patients, the mean values for eGFR declined across all treatment periods. Already Markello et al. describe a downward slope change in kidney function in cysteamine treated patients although not as steep as those untreated or partly treated [20]. In our study, the annual decrease in kidney filtration rate seemed more pronounced during the ER-treatment period. Whether this is due to a poorer disease control, disease progression or the single events in one patient leading to a significant drop in eGFR is impossible to determine. Bäumner et al. reported two patients with a deterioration in disease control reflected by increasing white blood cell cystine values and deterioration of kidney function under treatment with ER-cysteamine for a period of 9 months [21]. In contrast, Langman et al. and Ahlenstiel-Grunow et al. did not observe a change in eGFR over a 2-year and 1-year follow-up, respectively. However, both studies [6, 15] were conducted in a homogeneous patient group that included only children whereas our study included only a small number of patients with a very large age gap. Furthermore, the follow-up setting by Langman et al. targeted patients with an optimal disease control with WBC cystine levels always below 1 nmol hemicystine/mg protein which was not achieved on average in our real-life setting. Nevertheless, the distinct reasons for the downward trend in the change of eGFR should be pursued in further investigations.

The individual height *Z*-score shows a trend towards a positive development after switch. However, due to the heterogeneity of the group regarding puberty status and eGFR, it is hard to draw a definitive conclusion.

The number of reported ADRs related to cysteamine treatment was rather low in our study. Not every patient was affected. It should be noted, due to the retrospective study design with a pre-defined data collection period before switch, some ADRs and AEs starting on IR-cysteamine, but before the defined treatment period, could have been overlooked. None of the side effects were unexpected considering the side effect profile of cysteamine [8, 9] and the symptoms associated with the nephropathic cystinosis itself [15, 16]. The most common ADRs were of gastrointestinal origin. In both periods, the ADRs were mostly of mild severity. The absolute number of events was higher during the ER-treatment which is in line with the findings of Langman et al. [16], who also reported more gastrointestinal side effects under ER-cysteamine. More recent short-term studies, though, report fewer or no side effects during ER-treatment [6, 19].

The cysteamine metabolites dimethyl sulphide and methanethiol result in halitosis and bad body odour, side effects substantially influencing patients’ quality of life as well as adherence to treatment especially with adolescent patients. In agreement with published data [6, 13, 19], 4 patients also showed the benefit of less halitosis during our long-term treatment. But data are inconclusive, with 2 other patients reporting worsening of symptoms.

There were several limitations to our study. First, it must be taken into consideration that our data comes from a small, heterogeneous, patient group which varied in age from 1.8 to 36.0 years at switch. Furthermore, as nephropathic cystinosis is a rare disease, an uncontrolled study design was the only feasible design for this non-interventional retrospective study. Accordingly, the study carries the general limitations inherent to conduction without a control group. Due to the real-life design in the normal clinical setting, patients did not perform assessments at the same time and at the same number which means that time periods considered and number of measurements leading to the individual mean values differ from patient to patient. It must also be taken into consideration that some patients in our study did not meet the criterion for a well-controlled disease due to a lack of adherence. In addition, the disease progression over time was expected to have a negative impact for certain measures in the ER-treatment period, such as kidney function. However, the examination of individual patients and the course of the disease under the different forms of therapy and the comparison of small groups provide important indicative findings for the treatment of cystinosis regarding the efficacy and safety resulting from the therapy.

In conclusion, our long-term findings confirm that switching from IR- to ER-cysteamine is a feasible and well-tolerated option in routine clinical practice. Our study did not directly assess patients’ quality of life. However, the study has provided further indications that patients could benefit from less halitosis and that the twice-daily dosing may lead to better treatment adherence. Furthermore, long-term follow-up studies and observations are needed to provide more data regarding the long-term effects of ER-cysteamine on disease control reflected by cystine levels and kidney function.

### Supplementary information

Below is the link to the electronic supplementary material.

Graphical abstract (PPTX 48 KB)
