# Supplementary material for: The effects of transitioning from immediate release to extended release cysteamine therapy in Norwegian patients with nephropathic cystinosis: a retrospective study
Source: Pediatr Nephrol. 2023 May 23;38(11):3671–9. doi: 10.1007/s00467-023-06005-w (PMC10514171; doi:10.1007/s00467-023-06005-w)
Supplement: Supplementary file 1 — Graphical abstract (PPTX 48 KB) [file 467_2023_6005_MOESM1_ESM.pptx]

## Slide 1
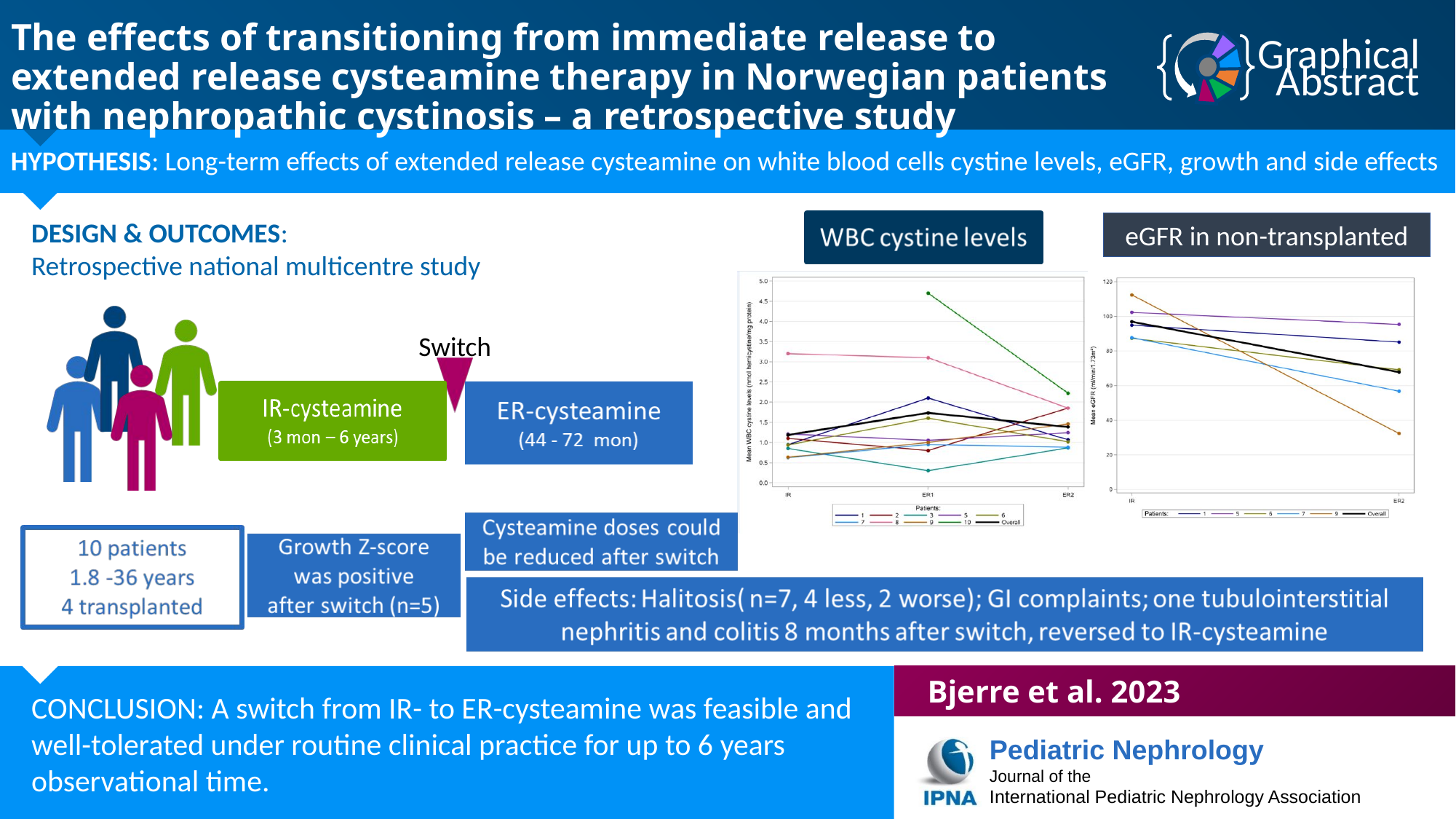

The effects of transitioning from immediate release to extended release cysteamine therapy in Norwegian patients with nephropathic cystinosis – a retrospective study
HYPOTHESIS: Long-term effects of extended release cysteamine on white blood cells cystine levels, eGFR, growth and side effects
DESIGN & OUTCOMES:
Retrospective national multicentre study
eGFR in non-transplanted
Switch
Bjerre et al. 2023
CONCLUSION: A switch from IR- to ER-cysteamine was feasible and well-tolerated under routine clinical practice for up to 6 years observational time.
